# Early arthroscopic release in stiff shoulder

**DOI:** 10.4103/0973-6042.40455

**Published:** 2008

**Authors:** Dhananjaya Sabat, Vinod Kumar

**Affiliations:** Department of Orthopedic Surgery, Maulana Azad Medical College and Associated LN Hospital, New Delhi, India

**Keywords:** Arthroscopic release, stiff shoulder

## Abstract

**Purpose:**

To evaluate the results of early arthroscopic release in the patients of stiff shoulder

**Methods:**

Twenty patients of stiff shoulder, who had symptoms for at least three months and failed to improve with steroid injections and physical therapy of 6 weeks duration, underwent arthroscopic release. The average time between onset of symptoms and the time of surgery was 4 months and 2 weeks. The functional outcome was evaluated using ASES and Constant and Murley scoring systems.

**Results:**

All the patients showed significant improvement in the range of motion and relief of pain by end of three months following the procedure. At 12 months, mean improvement in ASES score is 38 points and Constant and Murley score is 4O.5 points. All patients returned to work by 3-5 months (average -4.5 months).

**Conclusion:**

Early arthroscopic release showed promising results with reliable increase in range of motion, early relief of symptoms and consequent early return to work. So it is highly recommended in properly selected patients.

**Level of evidence:**

Level IV

## INTRODUCTION

Frozen shoulder is a common problem in our clinics and classically considered as a self-limiting condition.[1,2] Stiffness is poorly tolerated when limitations interfere with athletics, daily activities and hygiene. Despite years of investigations, little agreement exists regarding its diagnosis, etiology, pathology and management. 

The current working definition describes the disease as “a condition of uncertain etiology characterized by significant restriction of both active and passive shoulder motion that occurs in the absence of a known intrinsic shoulder disorder.”[3] It is usually divided into primary and secondary forms. In the primary form there is no associated disease or a history of trauma, whilst the secondary form occurs after trauma or surgery.

The initial management of stiff shoulder consists of encouragement of gentle physical therapy, performed either independently or in a supervised setting. The majority of patients will respond to non-operative treatment and require no further treatment. But when this fails to improve with non-operative treatment, the further management path is controversial. Suggested treatment regimens include continued physical therapy, steroid injections, hydraulic distension, manipulation under anesthesia and surgical release - open or arthroscopic. Now arthroscopic release is a well-established procedure recommended for refractory shoulder stiffness when all other management procedures fail.[4–6]

Still controversy exists regarding the timing of arthroscopic release. Failure of six months of conservative treatment is the most accepted indication of arthroscopic release.[4,7] But with established safety of the procedure and predictable outcome, early arthroscopic release can be recommended in properly selected patients, e.g. patients with marked disability and high functional demands.[8] Some authors suggested that surgical treatment can accelerate recovery in more serious cases.[4]

The purpose of this study is to evaluate results of early arthroscopic release in selected patients of stiff shoulder.

## MATERIALS AND METHODS

During the period of October 2004 to March 2006, a total 93 patients were diagnosed in our clinics with stiff shoulders as per the criteria suggested by Zuckerman *et al.*[9] (1) insidious onset of true shoulder pain (2) night pain (3) painful restriction of both active and passive elevation to less than 100 degrees and / or external rotation to less than one half of normal and (4) normal radiologic appearance.

Out of these, 20 patients (21.5%) who didn't show improvement in range of motion and pain with at least three months duration of symptoms, which included at least 6 weeks of aggressive physiotherapy and intraarticular steroid injections (40 mg Triamcinolone by posterior route) given at interval of two weeks (maximum 3), were included in the study.[8] The study does not include patients with degenerative changes in shoulder, patients who cannot tolerate the surgical stress of a fluid challenge (e.g. renal failure or cardiac failure), patients unable to understand or cooperate with a stretching motion program and patients with personality disorders.[7]

The age of the patient's undergone arthroscopic release ranged from 37 to 66 years (mean 53.65 years) [Table 1]. The majority of cases were males (12 males and 8 females). All patients were right dominant. Left side was involved in 10 whereas right side was involved in 9 patients. One patient had bilateral involvement in which only right side was operated upon. Three patients (2 males and 1 female) were suffering from NIDDM (Non Insulin Dependent Diabetes Mellitus). Blood sugar levels were uncontrolled at the presentation, which was controlled prior to surgery and maintained in the follow-up. One patient with NIDDM had bilateral involvement. All patients belong to occupations demanding high physical activity; including manual laborer, plumber, bus driver, tailor etc. The average duration of symptoms prior to surgery was 4 months and 2 weeks (range= 3-6 months).

**Table 1:** Patient characteristics

		Total	Male	Female
Patients		20	12	8
Etiology	Idiopathic	16	10	6
	Posttraumatic	1	-	1
	NIDDM	3	2	1
Side involved				
	Right	9	5	4
	Left	10	4	6
	Bilateral	1	1	-
Side operated				
	Right	10	6	4
	Left	10	4	6
Mean Age		53.65	54.2	53.1
Duration of symptoms prior to surgery (average in months)		4.42	4.01	4.9

The surgery was performed under general anesthesia with patient positioned in lateral decubitus position. First the range of motion was recorded in both the shoulders under anesthesia. A traction device with 5 kg weight was attached to the involved side to keep the arm at 30° abduction. After proper cleaning and draping, the bony landmarks were identified and marked. Then a standard posterior portal was established at the posterior soft spot approximately at 2 cm medial and 2-3 cm distal to the posterolateral tip of the acromion with rounded trocar and cannula. Whenever the entry was not possible at the standard posterior portal soft spot, we moved the trocar superiorly to make an entry among superior glenoid, rotator cuff and the humeral head where joint space is wider. 1.5% glycine was used as medium.

Some cases (7 of 20) required manipulation in the form of gentle elevation in the scapular plane to make space for arthroscope. The involved limb was grasped proximally near the axilla to shorten the lever arm and the arm was gently elevated in the scapular plane. This maneuver was invariably associated with audible popping of the contracted joint capsule. This manipulation doesn't appear to involve the risk any iatrogenic injury though there is risk of more fluid extravasation.

Diagnostic arthroscopy was performed in a standardized sequence starting from the long head of biceps. The sites and severity of synovitis were noted. Then anterior portal was established by indirect method. The arthroscope was directed at the rotator interval and a spinal needle is inserted into the joint from a point 1 cm lateral and 1 to 2 cm cephalad to the lateral subcutaneous border of the coracoid process. After skin incision, a 5 mm cannula was placed through it.

Rotator interval synovitis was resected with a motorized shaver. Unipolar electrocautery with hooked tip was introduced and capsular release was performed sequentially. Then anterior capsule was released through anterior portal beginning just inferior to biceps tendon and continuing to the inferior edge of glenoid at a distance of 1cm from the glenoid rim up to 5 o’ clock position for right shoulder. Superior, middle and inferior glenohumeral ligaments were released. Subscapularis was not resected. Posterior capsule was released after switching the portals. Axillary pouch contracture was not released with electorocautery to avoid damage to axillary nerve; it was addressed in final manipulation. Residual synovitis was resected with motorized shaver.

Subacromial space was inspected and release of bursal adhesions was done if required. Finally shoulder was manipulated to evaluate and improve the motion achieved by arthroscopic release. A standard sequence of manipulation was followed-abduction and elevation, external rotation, internal rotation in maximum abduction, cross body adduction and finally behind-the-back internal rotation. The amount of force required to achieve full range of motion was minimal.

An intra-articular catheter was placed through the posterior portal for postoperative pain relief. Bulky dressing was done. A cuff and collar sling was given only for 24h. No intraarticular steroid was used because capsular resection will cause steroid to extravasate and lose its effectiveness.

Postoperative physiotherapy [Table 2] was started on the day of surgery.[2] Patients were kept in hospital for 72h for active assisted physiotherapy. 20 ml of 0.25% Bupivacaine was instilled every 8 hourly through the intra-articular catheter for pain relief. The catheter was removed after 48h. The rehabilitation protocol followed is tabulated in Table 2.

**Table 2:** Rehabilitation schedule:[2]

**Day 1** Pendular exercise, Supine - assisted active flexion and external rotation at 0° with a stick
**Day 2-4** Stretches- Range of motion exercise 10 times every 2 hours. Encourage use of heat. Continue with above.
**Day 4 -10** Stretches 10 times 6 hourly; heat and massage as required. Add external rotation with a stick at 45°, 90° and 135° of abduction as tolerated.
**Day 10 - 2.5 weeks** Add in supine- extension, short lever to long lever progression as tolerated; standing- assisted horizontal flexion and extension in 0° of abduction with a stick. Accessory glide mobilization technique in neutral may commence. Isometric rotator cuff and scapula stabilizer exercises.
**2.5-3.5 weeks** Add in supine- assisted horizontal flexion at 0° and 45° of flexion. Standing - assisted hand behind back.
**3.5-4 weeks** Add in supine horizontal flexion at 90° of flexion/internal rotation at 90° of abduction. Isotonic rotator cuff and scapula stabilizer exercises.
**4 weeks** onwards Individual program modified and continued until maximal range of motion achieved and good functional strength requirements depending on individual's functional, occupational and sporting requirements.

Pain was assessed on a 10 cm Visual analog Scale. Passive movements of both affected and non-affected shoulders were obtained with a goniometer. Clinical evaluation based on ASES score[10] and Constant and Murley score[11] was done at regular intervals (two weekly for first three months and then at three month intervals). The average duration of follow-up was 18 months (range - 14 - 24 months). Statistical analysis was performed with the *Chi square* test, repeated measure ANOVA and Friedman's test (for nonparametric data).

## RESULTS

All cases manifested reduced intraarticular volume (measured by measuring amount of fluid that can be injected to the joint prior to surgery) and highly vascular infolding of the synovium. The rotator interval was most affected with proliferative synovitis. The subscapularis recess was obliterated with proliferative synovium. Long head of biceps was found to have proliferative synovitis (10 cases) and patchy, granular, matted area of granulation tissue (6 cases) which was resected with shaver. The axillary recess appeared contracted. Coracohumeral ligament appeared thick and cordlike (8 cases). Incomplete rotator cuff tear was seen in one case. Subacromial adhesions was seen in three cases; 1 case was post-traumatic, 1 having a chronic rotator cuff tear, the cause in the third was not found. After capsular release, capsular thickening was noted in all.

All patients showed improvement in symptoms following surgery. No procedure related complication occurred. Some patients (5 of 20) showed transient flaring up of pain in early postoperative period, which improved with time. 80% of the patients achieved pain-free range of motion by the end 12 weeks and the improvement was maintained till the last follow-up. Rapid improvement was seen in abduction and external rotation, whereas internal rotation was more resistant to recover. By 6 months, 95% patients were pain free and able to lie on the affected side comfortably and 80% were able to place 1lb weight on a shelf at shoulder height. All patients returned to work by 3 - 6 months (average - 4.5 months) and 85% could pursue their previous occupation. The remaining three patients changed to a job which was less demanding physically. The improvement in six ranges of motion, Visual Analog Pain scale, Constant and Murley score and ASES score at the end of one year of follow-up are significant (p value< 0.001) [Table 3]. At 12 months, mean improvement in Visual Analog Pain scale is 5.25 points, ASES score is 38 points and Constant and Murley score is 4O.5 points. The gain in range of motion was maintained and no recurrence was seen till last follow-up.

**Table 3:** Results of early arthroscopic release

Mean ROM ± SD	Preop.	6^th^ week	6^th^ month	1 year	Change in means ± SD	*p* Value
Abduction	86.75±6.18	136.85±11.9	151.75±13.8	157.75±14.7	71±17.23	< 0.001
Foreword flexion	83.2±6.09	127.65±11.8	141.45±14.8	147.35±15.5	64.15±16.89	< 0.001
External rotation	11.25±4.36	36.4±10.6	48.3±12.8	53.25±13.4	42±14.85	< 0.001
ER at 90° abduction*	15.65±4.89	47.35±11.4	61.95±12.6	68.65±12.9	53±14.27	< 0.001
Internal rotation	Gluteal	L5	L2	D12	8 spinous levels	--
IR at 90° abduction*	10.05±2.06	27.85±5.95	37.95±6.76	43.05±8.31	33±7.97	< 0.001
VAS	6.8±0.768	5.7±0.979	3±0.795	1.55±1.08	5.25±1.32	< 0.001
C and M score	22.5±1.70	34.8±6.14	55.8±6.22	63±6.74	40.5±6.91	< 0.001
ASES score	20.8±1.58	31.15±5.77	51.65±6.20	58.8±6.27	38±6.36	< 0.001

* = when abduction < 90° ROM was measured at maximum abduction

Functional results were analyzed according to the rating criteria proposed by Pollock *et al.*[5] [Table 4]. Results were excellent in nine cases (45%), satisfactory in 10 cases (50%) and unsatisfactory in only one case (5%). The patient who had an unsatisfactory outcome had persistent stiffness affecting overhead activities though the pain relief was satisfactory.

Results were not affected by age, sex, side involved or duration of symptoms prior to surgery (p value > 0.05) [Table 5].

**Table 4:** Rating Criteria[5]

	Excellent	Satisfactory	Unsatisfactory
Motion			
(FE, ER, IR)	170°/50°/D10	160°/40°/L1	FE<140°
Function	Unlimited	Satisfactory	Limited
Satisfaction	Yes	Yes	No
Pain	None	Slight	Moderate to severe

**Table 5:** Functional results and relation with probable prognostic factors

		Excellent	Satisfactory	Unsatisfactory	*P* -value
Total		9	10	1	---
Age	< 55 years	5	5	-	0.574
	> 55 years	4	5	1	
Sex	Male	5	6	1	0.626
	Female	4	4	-
Duration of symptoms (in months)	3 - 4. 5	5	6	0	0.574
	4.5 - 6	4	4	1	
Side					
operated	Left	5	5	-	0.574
	Right	4	5	1	

## DISCUSSION

Most of the authors recommend at least six months of conservative treatment prior to capsular release.[1,12] Some authors explained that frozen shoulder is an algoneurodystrophic process and normally surgery is contraindicated during the acute phase of reflex sympathetic dystrophy. So, surgery should be performed after a suitable waiting time if patient still have restriction of motion and functional impairment.[13] The minimum duration of conservative treatment prior to arthroscopic release was described by Ide and Takaji, which included at least six weeks of conservative treatment without progress and symptoms for at least three months.[8]

Neer stated that the presence of disability depends on the functional demands of the patient.[4] Most patients cannot tolerate a debilitating chronically painful extremity during productive years of life and are concerned about the possibility of developing permanent dysfunction.[7] Some patients may be unwilling to wait the time required for the resolution of symptoms.[14] Older patients, who have less functional demands, can tolerate more restrictions in any plane. This may be due to an adaptation to the restriction or to the fact that the restriction in planes that are unimportant for the activities of daily living. But for a young active patient, 150 degrees of active elevation, 50 degrees of external rotation and internal rotation to the level of eighth thoracic vertebra is probably sufficient for normal function.[4] Also the psychosocial and economical effects due to the disease must be taken in to consideration. To conclude, patients’ personality, functional demands, compliance with therapy and progress with therapy must be properly evaluated before planning for surgery.[15]

The entry to the joint was found to be difficult. Some patients needed prior manipulation in form of gentle elevation in the scapular plane to make space for the arthroscopy.[16] The hooked tip unipolar cautery was able to ablate a swathe of capsule and allowed for a controlled, less traumatic and complete release. Less force was required for the final manipulation itself. Using consistent technique and aftercare, arthroscopic capsular release procedure allowed us to treat the cases. Treatment failures were not seen, which may be due to proper resection of pathologic synovium, controlled capsular release and good postoperative physiotherapy programme. We agree with Nicholson that the natural history of this poorly understood condition was possibly shortened because patients attained final pain-free motion within an average three months.[15]

Several risk factors have been thought to reduce the possibility of satisfactory outcome. We were unable to identify age, sex, side of involvement and the duration of symptoms at the time of presentation as the risk factors affecting the subjective or objective outcome. Due to the strict selection criteria used in our study, the cases included represent the worst spectrum of the disease. So no conclusion can be derived about the effect of severity of symptoms prior to surgery on the outcome.

The high degree of success obtained in patients with primary stiff shoulder contrasts greatly with the results obtained in diabetic patients (IDDM) with stiff shoulder.[5] Diabetics have been reported to develop recalcitrant forms of shoulder stiffness.[7] In our study, three patients had NIDDM. Though initial improvement was poor when compared to patients without diabetes; the end results were comparable. No recurrence of symptoms was seen.

Earlier studies show that patients with associated intraarticular pathology usually show considerably poorer result.[15] Partial tear of rotator cuff is considered as a poor prognostic factor.[12] In our study, one patient had partial thickness rotator cuff tear who had satisfactory outcome (cuff tear was not repaired during surgery).

All patients in this series were compliant with both their postoperative exercises and physiotherapy. We believe that a specifically designed physiotherapy programme addresses muscle tightness and shoulder atrophy associated with this condition. Failure to follow such a regimen may compromise results.

The management of secondary stiffness still remains controversial.[4,6] Some authors obtained good results by arthroscopic treatment regardless of etiology,[4,5,6,17] but others reported that there was less improvement in the secondary stiff shoulder.[18,19] Subacromial bursoscopy should be performed in all posttraumatic stiff shoulder to evaluate and address the rotator cuff pathology.[20] In our study, we had only one case of posttraumatic stiff shoulder, who had excellent recovery.

The intraarticular catheter placement and Bupivacaine instillation was found to be safe and effective method of postoperative pain control and helped in starting physiotherapy early.[21] The requirement of oral or parenteral analgesics was drastically less. This also precluded the use of repeated interscalene block or placement of interscalene catheter for pain relief.[5,22] There are few animal experimental reports suggest continuous intraarticular catheter with bupivacaine infiltration with or without epinephrine to be chondrotoxic. This toxicity is dose and time-dependent.[23] But after capsular release, this toxicity might be less due to easy extravasation of Bupivacaine.

There are a few limitations in the study. The number of patients included were small due to the strict inclusion criteria. Majority of the cases operated upon were masculine which may not be representing the population as the sample size was small. Also elderly females in this country are more reluctant to undergo surgical treatment, which may have affected the study group. The follow-up period is 18 months on an average (range 14-24 mo). A long-term follow-up may be more helpful.

## CONCLUSION

Non-operative treatment still remains the first line of treatment of frozen shoulder; but when it fails arthroscopic release is a safe and viable option even when performed in the early inflammatory stage.
